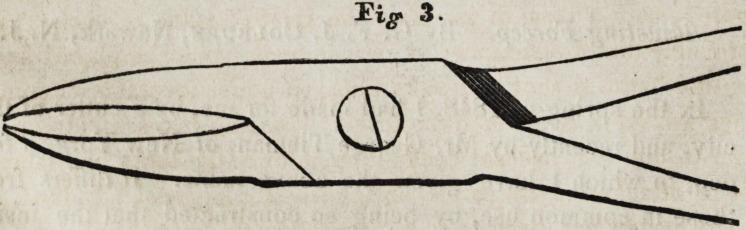# Adjusting Forcep

**Published:** 1851-10

**Authors:** G. F. J. Colburn

**Affiliations:** Newark, N.J.


### ARTICLE XI.

*Adjusting Forcep.* By G. F. J. Colburn, Newark, N. J.

In the spring of 1848, I had made for me, by a cutler of this city, and recently by Mr. George Tieman, of New York, a forcep, to which I have given the above name. It differs from those in common use, by being so constructed that the inside beam of the instrument can be lengthened, making one beak longer than the other; the object of which is, to enable the operator to grasp a tooth or root that has the inside or outside wall broken or decayed below the line of the gum, by adjusting the beaks of the forcep, so that both are brought to bear on the firm bone, as is shown in figures 1 and 2.

Fig. 1

Fig. 2.

The beak is adjusted by turning the head of the rivet (which is left large for this purpose) and removing it, then pushing up the inside beam, which has a notch cut out of it, at the junction of the handles and joint, until the second hole is ready for the rivet, which put in its place, the inside beam is now so elongated, that the beak will reach the firm bone beyond the decay. By reversing the instrument, the same beak will answer for the outside, or both beaks can be made of equal length, as in the common forcep.

Fig 3.

The one instrument combining the uses and advantages of three separate ones. This forcep will be found to be the most useful in the extraction of incisors and bicuspids. It can be used with or without Dr. Hullihen's improvement.

The difference in its construction from the forcep in general use, is, simply having two holes instead of one, in the joint part of the inside beam for the rivet, and a notch cut out of the beam at the juncture of the handle and joint, sufficient to give the beak the required length, the small end of the rivet has a thread cut on it and a corresponding one in the rivet hole of the other joint, for the purpose of retaining the rivet when screwed in, the other end of the rivet has a large head, so that it may be readily turned with the thumb and finger. I have used this forcep for nearly three years, and it has answered so good a purpose in lessening the embarrassment in extracting teeth, that I deem it my duty to lay it before the profession.

## Figures and Tables

**Fig. 1 f1:**
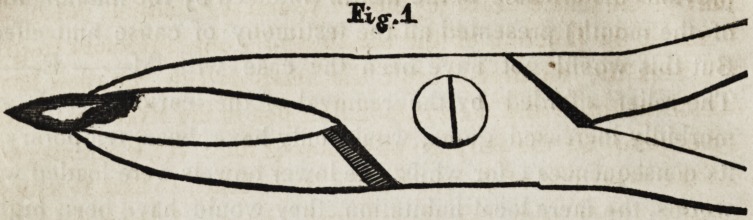


**Fig. 2. f2:**
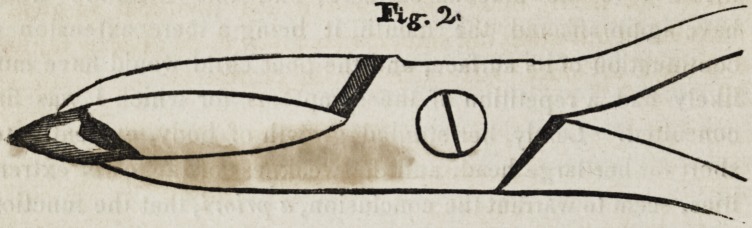


**Fig. 3. f3:**